# The expansion of activated naive DNA autoreactive B cells and its association with disease activity in systemic lupus erythematosus patients

**DOI:** 10.1186/s13075-021-02557-0

**Published:** 2021-07-06

**Authors:** Kittikorn Wangriatisak, Chokchai Thanadetsuntorn, Thamonwan Krittayapoositpot, Chaniya Leepiyasakulchai, Thanitta Suangtamai, Pintip Ngamjanyaporn, Ladawan Khowawisetsut, Prasong Khaenam, Chavachol Setthaudom, Prapaporn Pisitkun, Patchanee Chootong

**Affiliations:** 1 Department of Clinical Microbiology and Applied Technology, Faculty of Medical Technology, Mahidol University, 999 Phutthamonthon Sai 4 Road, Salaya, Nakhonpathom, 73170 Thailand; 2 Division of Allergy, Immunology and Rheumatology, Department of Medicine, Faculty of Medicine, Ramathibodi Hospital, Mahidol University, 270 Rama 6 Road, Ratchathewi, Bangkok, 10400 Thailand; 3 Department of Parasitology, Faculty of Medicine, Siriraj Hospital, Mahidol University, Bangkok, Thailand; 4 Center of Excellence for Microparticle and Exosome in Diseases, Department of Research and Development, Faculty of Medicine, Siriraj Hospital, Mahidol University, Bangkok, Thailand; 5 Center of Standardization and Product Validation, Faculty of Medical Technology, Mahidol University, Bangkok, Thailand; 6 Immunology Laboratory, Department of Pathology, Faculty of Medicine, Ramathibodi Hospital, Mahidol University, Bangkok, Thailand; 7 Translational Medicine Program, Faculty of Medicine, Ramathibodi Hospital, Mahidol University, Bangkok, Thailand

**Keywords:** Systemic lupus erythematosus, Activated naïve B cell, DNA autoreactive B cell, Disease activity

## Abstract

**Background:**

Autoreactive B cells are well recognized as key participants in the pathogenesis of systemic lupus erythematosus (SLE). However, elucidating the particular subset of B cells in producing anti-dsDNA antibodies is limited due to their B cell heterogeneity. This study aimed to identify peripheral B cell subpopulations that display autoreactivity to DNA and contribute to lupus pathogenesis.

**Methods:**

Flow cytometry was used to detect total B cell subsets (n = 20) and DNA autoreactive B cells (n = 15) in SLE patients’ peripheral blood. Clinical disease activities were assessed in SLE patients using modified SLEDAI-2 K and used for correlation analyses with expanded B cell subsets and DNA autoreactive B cells.

**Results:**

The increases of circulating double negative 2 (DN2) and activated naïve (aNAV) B cells were significantly observed in SLE patients. Expanded B cell subsets and DNA autoreactive B cells represented a high proportion of aNAV B cells with overexpression of CD69 and CD86. The frequencies of aNAV B cells in total B cell populations were significantly correlated with modified SLEDAI-2 K scores. Further analysis showed that expansion of aNAV DNA autoreactive B cells was more related to disease activity and serum anti-dsDNA antibody levels than to total aNAV B cells.

**Conclusion:**

Our study demonstrated an expansion of aNAV B cells in SLE patients. The association between the frequency of aNAV B cells and disease activity patients suggested that these expanded B cells may play a role in SLE pathogenesis.

**Supplementary Information:**

The online version contains supplementary material available at 10.1186/s13075-021-02557-0.

## Background

Systemic lupus erythematosus (SLE) is a systemic autoimmune disease characterized by a loss of immunological tolerance. Recognition of self-antigens causes an abnormal autoreactive immune response resulting in B cell production of autoantibodies. The resulting immune complexes are deposited in multiple organs and cause severe inflammation, which is fatally damaged if delayed in treatment [1, 2]. The sensitive and specific biomarkers to the change of SLE disease activity will help predict the disease flares and be a useful tool for lupus care. Recently, several cytokines (IL-6, IL-8, and IL-18) have been shown a high sensitivity with SLE disease activity [3, 4]. However, the changes in cytokines could reflect from the concomitant infection in lupus patients. Thus, the identification of a biomarker that is very specific to lupus activity is needed.

The anti-nuclear antibody (ANA) is a hallmark of SLE. Among these autoantibodies in SLE, anti-dsDNA antibodies are the most extensively studied. They are found in approximately 50% of SLE patients and are a specific diagnostic marker [5, 6]. However, the association between circulating anti-dsDNA antibody levels and lupus activity varies significantly across different studies [6–10]. The increases in these antibody levels are associated with SLE exacerbations, whereas reports of reductions associated with clinical benefit are limited [11–14]. The delayed clearance of autoantibody, which has a half-life of up to 30 days (depended on isotype), will not reflect the current SLE disease activity [15]. Although anti-dsDNA’s specificity to SLE is better than cytokines, immunoglobulin’s slow dynamic changes are not an excellent biomarker to reflect SLE disease activity. The anti-dsDNA producing cells may better reflect the disease activity and specific to SLE than serum anti-dsDNA or cytokines. Therefore, identifying the B cell population that produced anti-dsDNA is crucial, which may be a sensitive and specific biomarker to SLE disease activity.

In addition to pathogenic autoantibodies, B cells play other essential roles in SLE. Autoreactive B cells are phenotypically heterogeneous. Genetic background, hormonal milieu, and antigen exposure all contribute to this diversity [16–18]. Both naive and antigen-experienced B cell populations are correlated with SLE disease activity [19, 20]. Expansion of aNAV B cells (with highly expressed transcription factor T-bet and CD11c) was shown in patients with moderate to severe flares in disease activity [19, 21]. This B cell subset includes high clonality of the autoantibody secreting cells (auto-ASCs), indicating defective selection at the transitional stage and preferential differentiation contributing to SLE pathogenesis [21]. Also, antigen-experienced B cells [including atypical memory (AtMs), double negative 2 (DN2), or age-associated B cells (ABCs)] which share canonical phenotype and are identified as CD24^-^CD20^hi^, IgD^-^CD27^-^, or CD19^+^CD21^low^ B cells are frequently detected in SLE patients [20, 22, 23]. The stimulation of these autoreactive B cells results in the production of anti-Ro, anti-Sm, and anti-RNP autoantibodies [20, 22, 23]. It remains controversial as to which subsets of B cells are autoreactive and secrete anti-dsDNA autoantibodies. Thus, more research regarding the autoreactive B cells contributing to SLE pathogenesis would help monitor disease activity and predict lupus flares.

In this study, we identified peripheral B cell subsets that were autoreactive against DNA. To assess these DNA autoreactive B cells’ contribution in SLE pathogenesis, we determined their frequencies in SLE patients and analyzed the correlations with disease activity.

## Methods

### Subjects and study design

SLE patients were enrolled from the Department of Medicine, Ramathibodi Hospital, Bangkok, Thailand. Blood from 38 SLE patients was studied to detect the frequency of B cell subsets (n = 20) and their DNA tetramer-binding B cells (n = 15) and to phenotype aNAV DNA tetramer-binding B cells (n = 3). Correlation analyses were done between total aNAV and aNAV DNA tetramer-binding B cells and clinical parameters [modified SLEDAI-2 K score, erythrocyte sedimentation rate (ESR), anti-dsDNA antibodies, complement 3 (C3), and complement 4 (C4) levels] (Additional file 1: Tables S1 and Additional file 2: Tables S2).

Inclusion criteria for study subjects were as follows: (1) a diagnosis of SLE based on Systemic Lupus International Collaborating Clinics (SLICC) criteria 2012 [24, 25], (2) presence of SLE clinical manifestations and receipt of treatment with immunosuppressive drugs, and (3) age above 18 years. Exclusion criteria were as follows: (1) presence of a syndrome overlapping with SLE, (2) presence of infection, and (3) a diagnosis of cancer. Disease activity was measured by the modified SLEDAI-2 K. Laboratory parameters (ESR, anti-dsDNA antibodies, C3 and C4 levels) were determined and correlated with aNAV B cells’ frequency. Characteristics of study subjects are presented in Additional files 1 and 2. Healthy donors (n = 19) were recruited as controls (HCs) in our study. Of total 19 donors, 11 subjects were assigned for analysis of frequency and phenotype of peripheral B cell subsets. Eight samples were taken for determination of DNA tetramer-binding B cells and the baseline levels of anti-dsDNA autoantibodies. The study was approved by the Ethical Committee of Mahidol University (approval number, MURA 2015/731).

### Tetramer generation

The DWEYSVWLSN peptide can behave as a dsDNA mimetope [26]. Immunization of BALB/c mice with an octameric form of this peptide results in the production of anti-dsDNA antibodies, which are involved in lupus nephritis by deposition in renal glomeruli [27–29]. Here, a DWEYSVWLSN peptide was used to identify DNA tetramer-binding B cells in SLE patients. A biotinylated peptide was synthesized (GenScript, NJ, USA) and used in tetramerization by combining 5 μl of biotinylated peptide stepwise in 1/2 volumes to 3 mM SA-R-phycoerythrin-PE at a molar ratio of 10:1 and incubating for 60 min at 4 °C.

### Tetramer enrichment

To define tetramer-stained B cells, PBMCs from SLE patients (n = 15) were resuspended to 100 μl in FACS buffer (PBS + 0.1% BSA) and incubated with anti-CD32 Fc blocker for 2 min. Next, PE-conjugated peptide tetramer was added at a concentration of 0.001 M and incubated for 15 min at 4 °C, and then washed in 2.5 ml cold FACS buffer. Tetramer-stained cells were resuspended to a volume of 100 μl FACS buffer per 10^6^ cells and then mixed with 10 μl anti-PE-conjugated magnetic MicroBeads. These tetramer-stained cells were enriched by positive selection with EasySep PE Positive Selection Kit (STEMCELL, Vancouver, Canada), incubated for 15 min at 4 °C, and washed with 2.5 ml FACS buffer. Finally, tetramer-enriched cells were stained with antibodies against B cell surface markers before flow cytometric analysis.

### B cell culture

The purified tetramer-binding B cells (2 × 10^2^) were seeded into 96-well plates to demonstrate tetramer-binding B cells’ capability that produces anti-dsDNA autoantibodies. These plates were pre-seeded overnight with CD154-expressing stromal cells (CD40L^Low^ cell line, a gift from Garnett H. Kelsoe) [30]. B cells were cultured with R848 (1 μg/ml) and calf-thymus DNA (10 μg/ml) in R5 medium (RPMI 1640 with 10% fetal bovine serum, 2 mM L-glutamine, 100 U/ml penicillin, 100 μg/ml streptomycin, 10 mM HEPES, 1 mM sodium pyruvate, and 1% MEM nonessential amino acids), supplemented with recombinant human IL-2 (50 ng/ml) and IL-21 (100 ng/ml) (PeproTech, NJ, USA) for 8 days. The supernatant from tetramer-binding B cell cultures was harvested and stored at − 20 °C for total IgG and anti-dsDNA antibody quantitation. DN2 B cells, representing pre-ASCs, were cultured as positive controls.

### Antibody staining and flow cytometry

To detect subpopulations and characterize phenotypes of B cells, PBMCs from SLE patients (n = 20) were separated and analyzed to determine the frequency of each B cell subset. Two × 10^5^ cells were resuspended in 50 μl FACS buffer and incubated with surface antibodies for 15 min at 4 °C. Antibodies used included FITC-CD19, APC-CD21, APC/Fire750-CD27, Alexa/fluorro700-CD11c, PerCP/Cy5.5-CXCR5, PerCP/Cy5.5-IgG, PE/Cy7-IgD, APC-HLA-DR, PE/Cy5-CD69, and PE/Cy5-CD86 (Biolegend, San Diego, USA). After staining, cells were washed with 1 ml FACS buffer and resuspended in 300 μl FACS buffer for surface marker analyses.

The frequency and phenotype of DNA tetramer-binding B cells were detected by the tetramer staining technique, resuspended in 100 μl FACS buffer, and incubated with surface antibodies for 15 min at 4 °C. The stained B cells were washed with 1 ml FACS buffer and resuspended in 500 μl FACS buffer for flow cytometric surface marker analysis. FACS data were acquired with a BD FACS Canto II (Becton-Dickinson Immunocytometry Systems, San Jose, USA) and analyzed with FlowJo software (v. 10.0; Tree Star Inc., CA, USA).

### Enzyme-linked immunosorbent assay (ELISA)

To detect anti-dsDNA and anti-peptide IgG antibodies in plasma or B cell culture supernatant, 96-well plates were coated with calf thymus DNA (10 μg/ml) or synthesized DWEYSVWLSNK peptide (20 μg/ml), and then held overnight at 4 °C. After blocking with HBS containing 10% FBS for 2 h, plates were incubated with subject plasma (diluted 1:200) or culture supernatant (undiluted) for 1 h at RT. Then, plates were washed five times with HBS containing MnCl_2_ and incubated with horseradish peroxidase (HRP)-conjugated, anti-human IgG antibodies (KPL, MA, USA) at a 1:1000 dilution for 1 h. Subsequently, plates were developed with tetramethylbenzidine (TMB) enzyme-substrate. After 15 min, the reaction was stopped by 1 N HCl and absorbance measured at 450 nm using a microplate reader (Bio-Rad, CA, USA). To detect total IgG, plates were coated with 1 μg/ml of IgG anti-human immunoglobulin (Mabtech AB, Stockholm, Sweden), and undiluted culture supernatant was added, followed by the addition of HRP-conjugated, anti-human IgG antibodies. The optical density (OD) of the reaction was measured at 450 nm after adding TMB substrate.

### Statistical analysis

Data were analyzed using GraphPad Prism software version 8 (GraphPad Software Inc., San Jose, CA, USA). Group comparisons were performed using two-tailed, unpaired Mann-Whitney U tests [presented as group median (interquartile range)]. The significance of correlations were calculated using Spearman’s correlation coefficient (*r*). Median ± interquartile range (IQR) was used for all statistical tests. Group differences with *p* values less than 0.05 were considered statistically significant.

## Results

### Increased proportion of aNAV and DN2 B cells in lupus patients

**Fig. 1:**
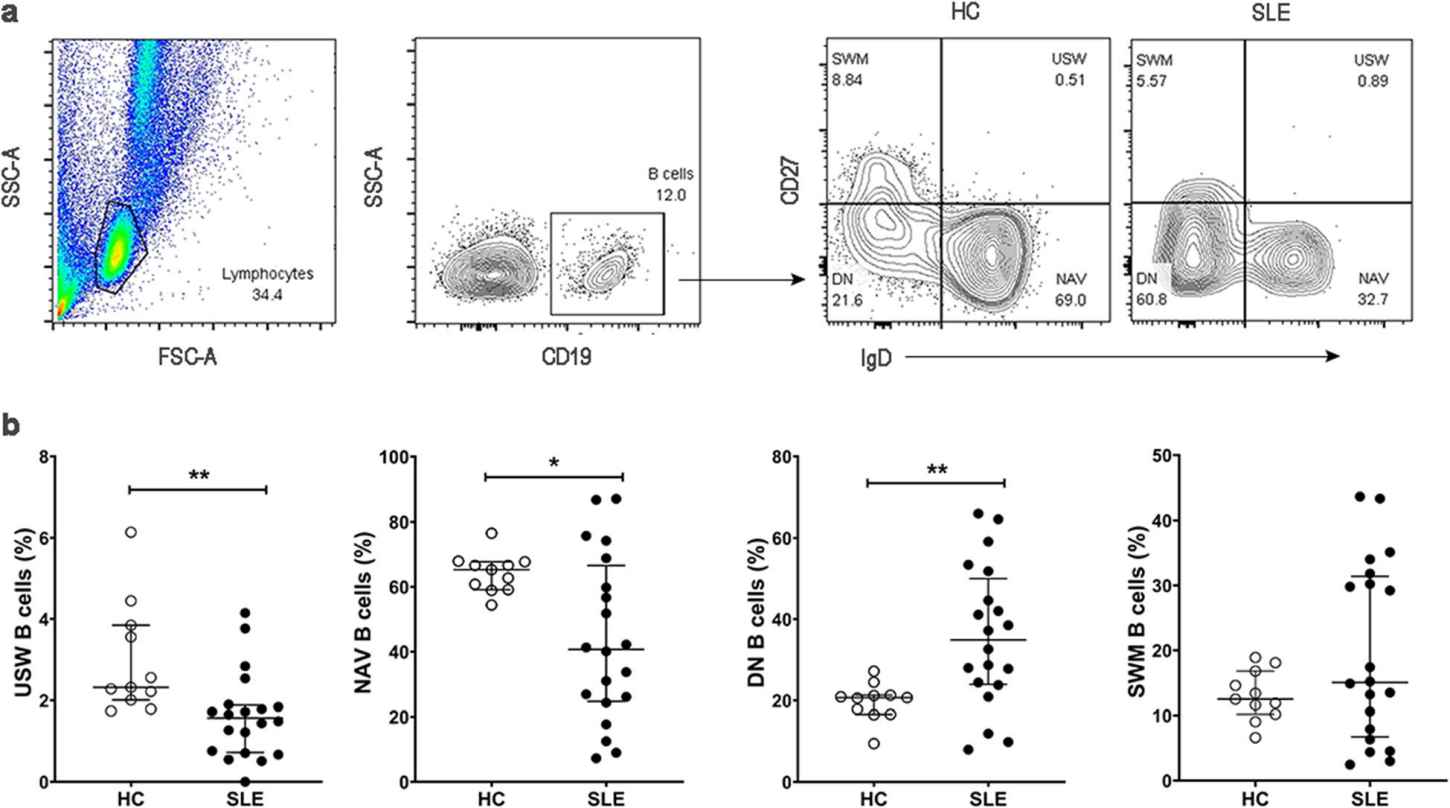
Phenotype and frequency of B cell subsets in SLE patients. **a** Representative gating strategy of CD19^+^ B cells from one SLE patient and one HC. The four independent subpopulations were gated: unswitched memory B cells (USW, IgD^+^CD27^+^), naïve B cells (NAV, IgD^+^CD27^-^), double negative B cells (DN, IgD^-^CD27^-^), and switched memory B cells (SWM, IgD^-^CD27+). **b** The frequency of USW, NAV, DN, and SWM B cells in SLE patients (n = 20) compared to HCs (n = 11). Bars represent median with interquartile range. p values were determined by the Mann-Whitney *U* test: *p < 0.05; **p < 0.01; ***p < 0.001; ****p < 0.0001

Four B cell subpopulations including switched memory (SWM; IgD^-^CD27^+^), unswitched memory (USW; IgD^+^CD27^+^), double negative (DN; IgD^-^CD27^-^), and naïve (NAV; IgD^+^CD27^-^) B cells were detected in the peripheral blood of SLE patients (Fig. 1a). The percentage of DN B cells was greater in patients than controls [34.90% (23.95–50.00%) vs 20.70% (16.60–21.30%), *p* < 0.01]. The frequencies of USW and naive B cells were lower in SLE patients than controls ([1.57% (0.72–1.89%) vs 2.32% (2.01-3.85%), *p* < 0.01] and [40.70% (24.75–66.55%) vs 65.30% (59.10–67.70%), *p* < 0.05], respectively). The frequency of SWM B cells did not differ significantly in SLE patients and HCs (Fig. 1b).

**Fig. 2:**
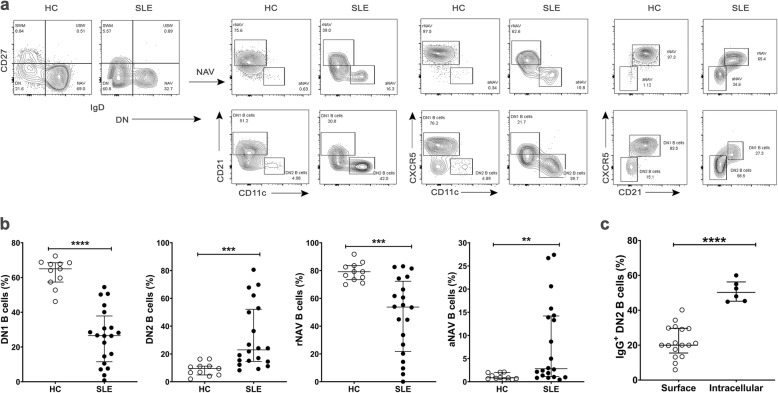
Subsets of double negative and naïve B cells. **a** Representative flow cytometry from SLE patient and HC. The NAV and DN B cells were classified and grouped as rNAV (CD21^+^, CD11c^-^, CXCR5^+^), aNAV (CD21^-^, CD11c^+^, CXCR5^-^), DN1 (CD21^+^, CD11c^-^, CXCR5^+^), and DN2 B cells (CD21^-^, CD11c^+^, CXCR5^-^). **b** The frequencies of DN and NAV B cell subsets in HCs (n = 11) compared to SLE patients (n = 20). **c** The frequency of intracellular IgG^+^ DN2 B cells (n = 6) as compared to surface IgG^+^ DN2 B cells (n = 17) in SLE patients. Bars represent median with interquartile range. *p* values were determined by the Mann-Whitney *U* test: *p < 0.05; **p < 0.01; ***p < 0.001; ****p < 0.0001

The expansions of DN and NAV B cell populations were further analyzed in SLE patients. Double negative 1 (DN1) and double negative 2 (DN2) B cells were distinguished based on their expression of CXCR5, CD21, and CD11c (Fig. 2a). We detected a higher frequency of CXCR5^-^CD21^-^CD11c^+^ DN2 B cells [22.95% (14.65–52.18%) vs 9.49% (4.88–11.20%), *p* < 0.001] and a lower frequency of CXCR5^+^CD21^+^CD11c^-^ DN1 B cells [26.55% (11.53–37.85%) vs 65.00% (57.40–68.50%), *p* < 0.0001] when compared with HCs (Fig. 2b). Naïve B cells, resting naïve (rNAV), and activated naïve (aNAV) B cells were defined based on CXCR5, CD21, and CD11c expression. The frequency of aNAV B cells was markedly greater in SLE patients than controls [2.80% (1.10–14.20%) vs 0.84% (0.64–1.99%), *p* < 0.01] (Fig. 2b). In contrast, the frequency of rNAV B cells was lower in patients than in HCs [53.75% (21.83–72.33%) vs 79.20% (73.50.10–83.60%), *p* < 0.001] (Fig. 2b). Interestingly, patients’ IgG expression levels within the cells of expanded DN2 B cells were higher than on the cell surfaces [intracellular: median = 50.25% (45.20–56.25%), surface: median = 20.00% (15.50–29.65%), *p* < 0.0001] (Fig. 2c).

### Binding reactivity of anti-peptide IgG and anti-dsDNA antibody production of tetramer-binding B cells

**Fig. 3:**
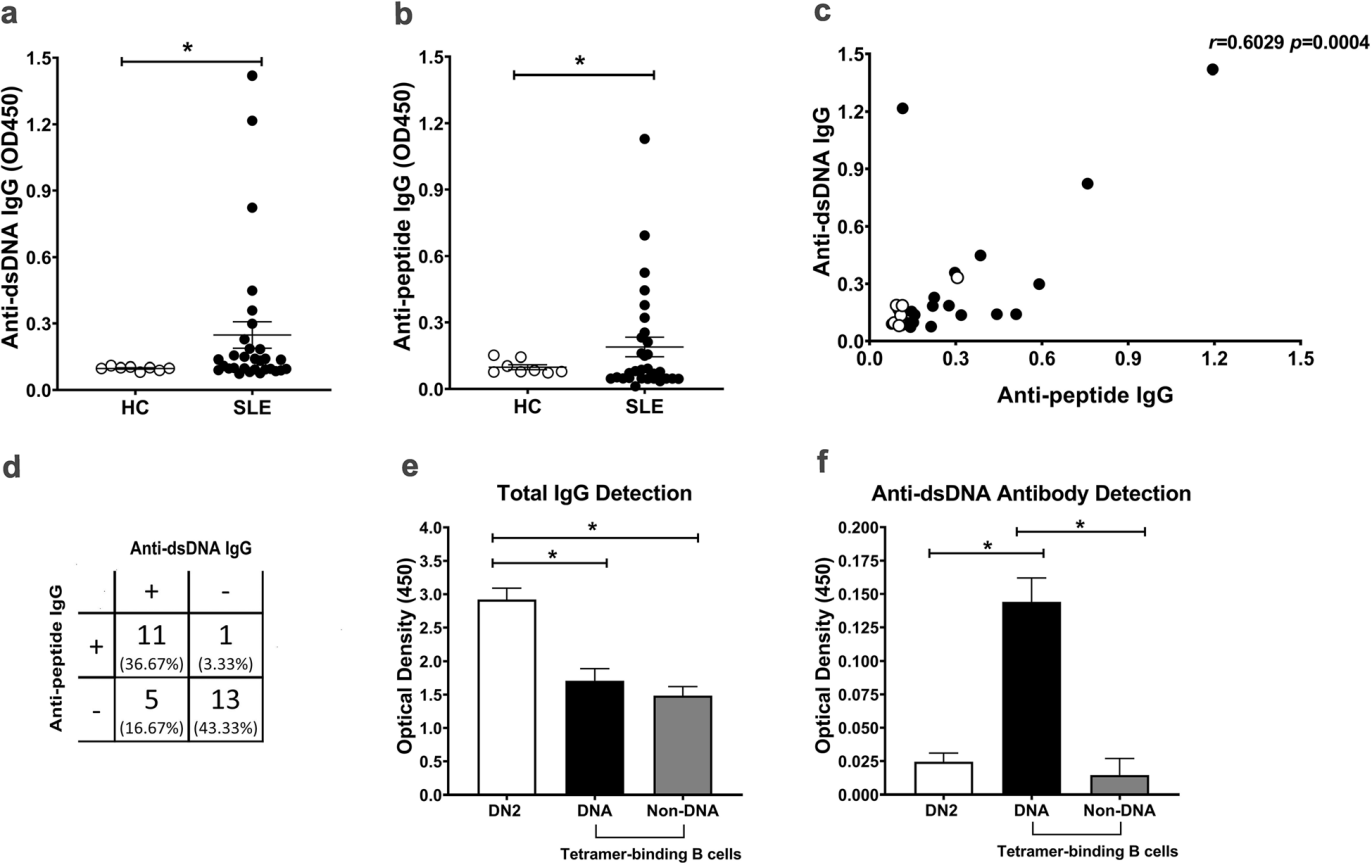
Characterization of anti-dsDNA and anti-peptide IgG reactivity. A significant difference exists between **a** anti-dsDNA IgG **b** anti-peptide IgG antibody levels in lupus patients (n = 30) compared with HCs (n = 8). **c** A significant correlation between anti-peptide IgG and anti-dsDNA IgG antibody levels in all individual patients. Healthy controls were shown in white (n = 8), lupus patients in black (n = 30). **d** The correlation analysis between anti-peptide and anti-dsDNA IgG levels in 30 SLE patients. Secretion levels of **e** total IgG **f** anti-dsDNA antibodies by DN2, DNA, and non-DNA tetramer-binding B cells detected after 8 days of in vitro culture (n = 3). Bars represent median with interquartile range. *p* values were determined by the Mann-Whitney *U* test: *p < 0.05; **p < 0.01; ***p < 0.001; ****p < 0.0001

To verify that the DWEYSVWLSN peptide can behave as a dsDNA mimetope [26], we first quantified the level of anti-dsDNA and anti-peptide IgG in serum SLE patients. Levels of anti-dsDNA IgG [0.248 (0.188–0.308) vs 0.098 (0.095.10–0.101), *p* < 0.05] and anti-peptide IgG [0.189 (0.145–0.233) vs 0.0985 (0.0874-0.110), *p* < 0.05] were significantly higher in subjects than controls (Fig. 3a, b). Furthermore, anti-dsDNA and anti-peptide IgG levels were significantly correlated (*r* = 0.6029, *p* = 0.0004, Fig. 3c). Of 30 SLE patients, 24 exhibited an accordance between anti-peptide and anti-dsDNA reactivity (80%; 11 samples were seropositive, and 13 samples were seronegative), whereas 6 patients showed a discordance between these antibodies (Fig. 3d).

Next, we demonstrated the anti-dsDNA producing cells using the tetramer binding DWEYSVWLSNK peptide. Both tetramer-binding and non-tetramer-binding B cells secreted total IgG antibodies. Higher anti-dsDNA antibodies were only detected in tetramer-binding B cell cultures (Fig. 3e, f). As expected, DN2 (a pre-ASC subset) produced high levels of total IgG (Fig. 3e, f).

### Relation of aNAV B cells to tetramer-binding B cells

**Fig. 4:**
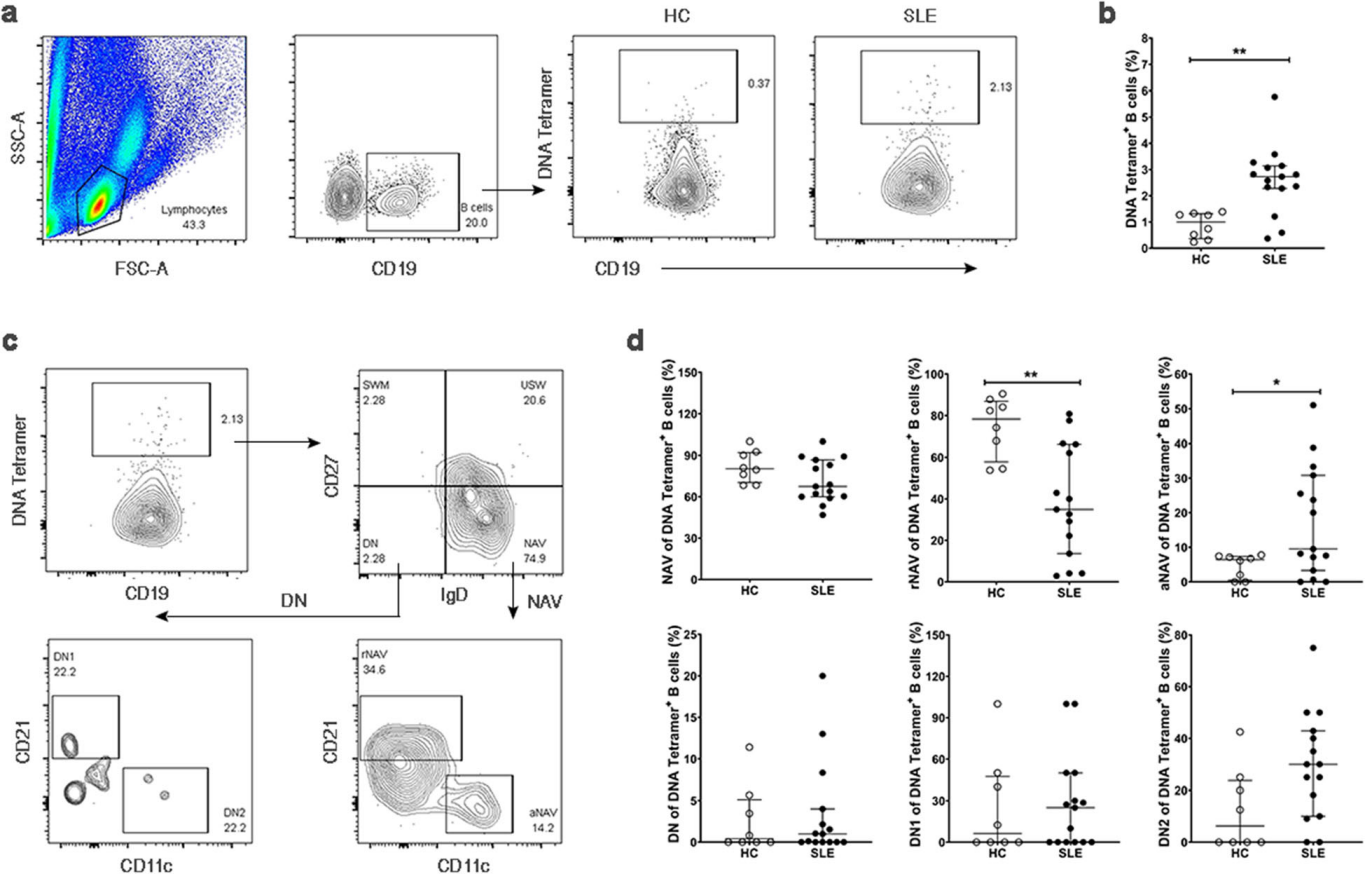
Identification and characterization of tetramer-binding B cells. **a** Gating strategy used to identify DNA tetramer-binding B cells. CD19^+^ B cells were gated, and the frequency of DNA tetramer-binding B cells was determined as shown in HCs and SLE patient. **b** The frequency of DNA tetramer-binding B cells in HCs (n = 8) compared to SLE patients (n = 15). **c** DNA tetramer-binding B cells were defined as double negative and naïve B cells; the NAV B cells were further classified into resting (rNAV) and activated (aNAV) naïve B cells; double negative B cells were classified into double negative 1 (DN1) and double negative 2 (DN2) B cells. **d** The comparative frequencies of naïve and double negative B cell subsets in HCs (n = 8) and SLE patients (n = 15). Bars represent median with interquartile range. *p* values were determined by the Mann-Whitney *U* test: *p < 0.05; **p < 0.01; ***p < 0.001; ****p < 0.0001

The frequency of tetramer-binding B cells in SLE patients was gated on CD19^+^ B cells (Fig. 4a). Tetramer-binding B cells were significantly more frequent in SLE patients than HCs [2.73% (2.28–3.14%) vs 1.00% (0.04–1.32%), *p* < 0.01] (Fig. 4b). Tetramer-binding B cells were further characterized to determine whether the expanded DN and NAV B cells were the DNA autoreactive cells. We found that most bound DNA autoreactive B cells were naïve B cells (IgD^+^CD27^-^) [67.40% (60.00–86.70%)], whereas they were present in lower proportions in DN B cells (IgD^-^CD27^-^) [3.41% (1.88–4.67%)] (Fig. 4c, d).

In addition, the phenotypic characterization of tetramer-binding B cells showed that aNAV B cells were more frequent in SLE patients than HCs [9.52% (3.29–30.80%) vs 6.42% (0.50–7.36%), *p* < 0.05], whereas rNAV cells were less frequent [34.90% (13.60–66.30%) vs 78.30% (57.85–86.88%), *p* < 0.01]. No significant difference was observed for DN B cells between DN B cell subsets of lupus patients and HCs (Fig. 4d). Of note, the activating profile, CD69, and CD86 were observed in an increased proportion of aNAV DNA tetramer-binding B cells compared with non-tetramer-binding, whereas no difference in the expression level of HLA-DR was observed between these B cell populations. (Additional file 4: Fig. S1).

### Association of expanded aNAV B cells with clinical parameters

**Fig. 5:**
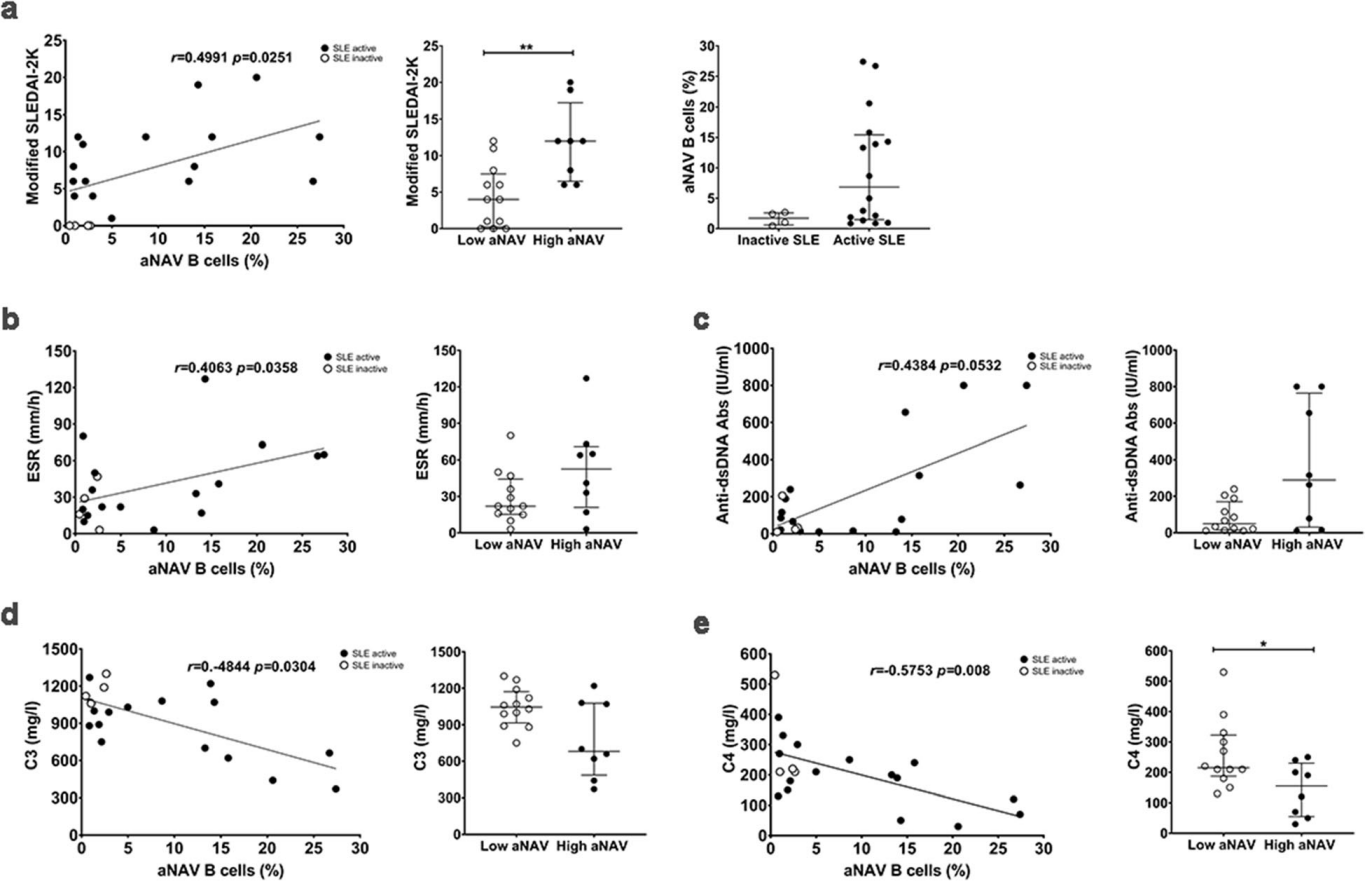
Correlation of aNAV B cells with clinical parameters. Correlation between the frequencies of aNAV B cells and clinical parameters in SLE patients (n = 20). **a** modified SLEDAI-2 K score (left panel), modified SLEDAI-2 K score in patients with an expansion of aNAV B cells (middle panel), and frequencies of aNAV B cells in inactive and active SLE patients (right panel), **b** ESR, **c** anti-dsDNA autoantibodies, **d** C3, and **e** C4 levels. Spearman’s correlation coefficient (*r*) was used as metric to calculate correlations between numerical data. Bars represent median with interquartile range. *p* values were determined by the Mann-Whitney *U* test: *p < 0.05; **p < 0.01; ***p < 0.001; ****p < 0.0001

Since aNAV B cells were found to be expanded in total CD19^+^ B cells and DNA tetramer-binding B cells of SLE patients, we hypothesized that the frequency of this subset correlated with clinical parameters and that aNAV B cells could be biomarkers for monitoring disease activity. Therefore, correlations between cell frequencies and clinically measured parameters (modified SLEDAI-2 K score, ESR, anti-dsDNA antibodies, C3 and C4 levels) were analyzed (Additional file 3: Table S3). The frequency of aNAV B cells was positively correlated with modified SLEDAI-2 K score (*r* = 0.4991, *p* = 0.0251). Significantly higher modified SLEDAI-2 K scores were observed in lupus patients with expanded aNAV B cells (Fig. 5a). In addition, the frequency of total aNAV B cells was positively correlated with ESR level (*r* = 0.4063, *p* = 0.0358, Fig. 5b), whereas no correlation was found with anti-dsDNA antibody level (Fig. 5c). These aNAV B cells were inversely correlated with C3 (*r* = -0.4844, *p* = 0.0304) and C4 levels (*r* = -0.5753, *p* = 0.008) (Fig. 5d, e).

**Fig. 6:**
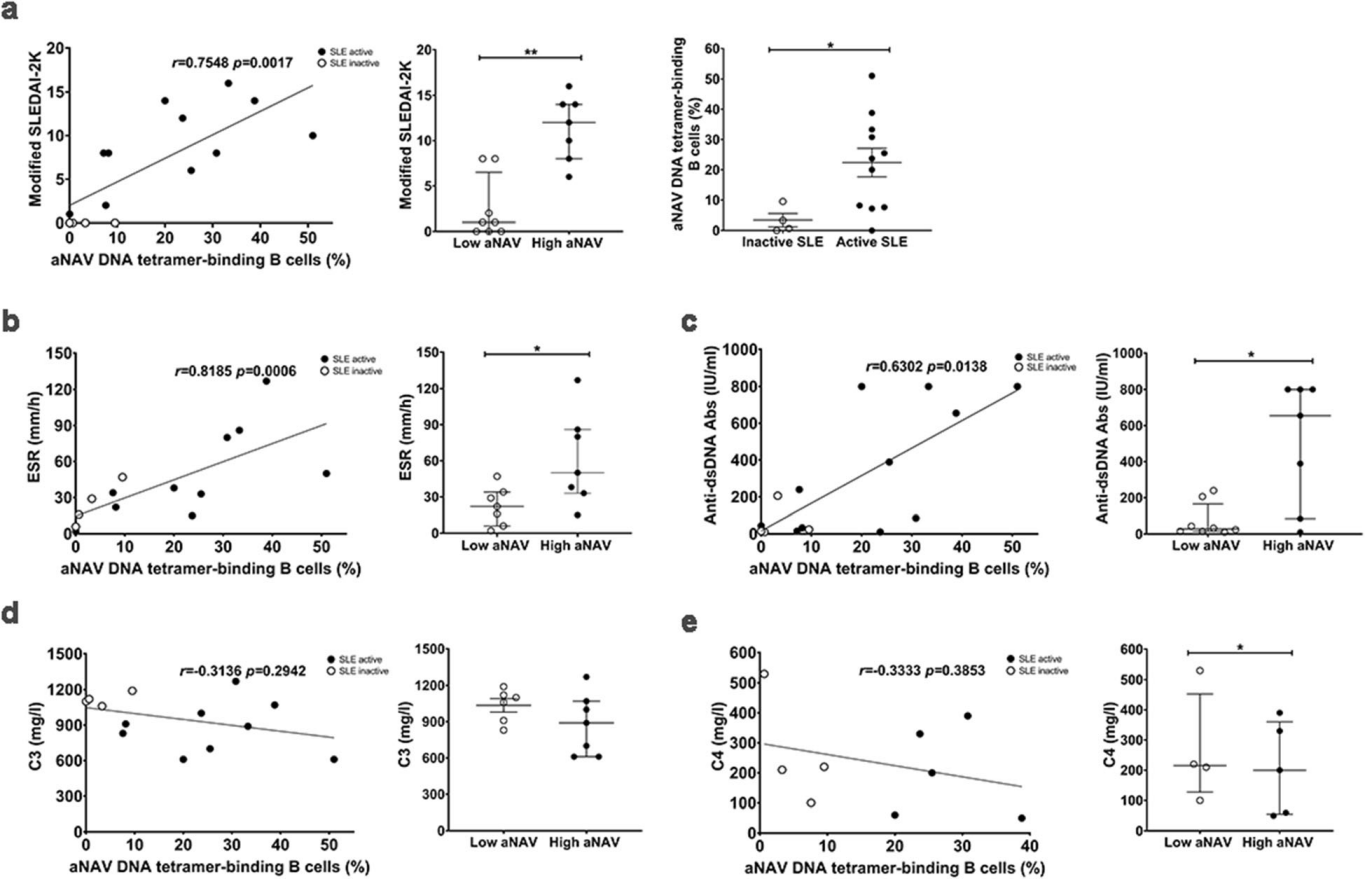
Correlation of activated naïve tetramer-binding B cells with clinical parameters. Correlation between the frequencies of aNAV DNA tetramer-binding B cells and clinical parameters in SLE patients. **a** modified SLEDAI-2 K score (left panel), modified SLEDAI-2 K score in patients with an expansion of aNAV DNA tetramer-binding B cells (middle panel), and frequencies of aNAV DNA tetramer-binding B cells in inactive and active SLE patients (right panel) (n = 15), **b** ESR (n = 14), **c** anti-dsDNA autoantibodies (n = 15), **d** C3 (n = 13), and **e** C4 (n = 9) levels. Spearman’s correlation coefficient (*r*) was used as metric to calculate correlations between numerical data. Bars represent median with interquartile range. *p* values were determined by the Mann-Whitney *U* test: *p < 0.05; **p < 0.01; ***p < 0.001; ****p < 0.0001

Correlation analysis of aNAV tetramer-binding B cell with clinical parameters, as described in Additional file 3, showed that the frequency of aNAV DNA tetramer-binding B cells was strongly (positively) correlated with modified SLEDAI-2 K score (*r* = 0.7548, *p* = 0.0017). Higher frequencies of these cells were observed in patients with greater disease activity and active SLE [23.70% (7.58–33.30%) vs 1.96% (0.16–7.96%), *p* < 0.05] (Fig. 6a). Moreover, the frequency of aNAV DNA tetramer-binding B cells was positively correlated with ESR (*r* = 0.8185, *p* = 0.0006) and anti-dsDNA antibody levels (*r* = 0.6302, *p* = 0.0138). Higher frequencies of these cells were observed in patients with high levels of ESR and anti-dsDNA antibody (Fig. 6b, c). However, no correlation was found between these B cells and C3, nor C4 levels in lupus patients (Fig. 6d, e).

## Discussion

Autoreactive B cells, found among the expansion of various B cell subsets in SLE patients, are considered as pathogenic since they recognize self-antigens and produce autoantibodies [20, 22, 23]. These cells contribute to the development of SLE and the activity of disease [19, 31, 32]. Such autoreactive B cells in SLE may provide a candidate biomarker for B cell therapeutic interventions. We found the expansions of DN2 and aNAV B cells in the blood from SLE patients. Within these expanded B cell subsets, aNAV B cells were mainly tetramer-binding B cells, and their antibodies mostly recognized DNA, suggesting their role in producing autoantibodies in SLE patients. Notably, the frequencies of aNAV B cells in both total and DNA autoreactive B cells were positively correlated with SLE disease activity. Altogether, our data suggested that the expansion of these B cells plays a pathogenic role in SLE by the active autoantibody production.

We demonstrated that SLE patients had high frequencies of DN2 and aNAV B cells. The aNAV B cells strongly correlated with modified SLEDAI-2 K score and ESR level, indicating that aNAV B cells may exhibit pathogenic functions and are associated explicitly with SLE progression. In explaining the possible pathogenic functions of aNAV B cells in development of anti-dsDNA autoantibody, it was demonstrated that DN2 and aNAV B cells shared a canonical phenotype characterized by CXCR5^-^CD21^-^CD11c^+^ expression. This suggests that DN2 and aNAV B cells originate and develop from resting naive B cells [20]. Recently, both DN2 and aNAV B cells were demonstrated to have the capacity to differentiate into ASCs and produce autoantibodies [20, 21]. Upon stimulation of naive B cells with TLR-7, IFN-γ and IL-21, these aNAV B cells can directly differentiate into DN2 B cells and plasma cells at days 3 and 5, respectively, indicating that DN2 B cells exhibit a pre-ASC stage of pathogenic relevance to autoantibody production [20, 33]. In addition, in cooperation with helper T cells in response to antigen stimulation, it could enchance autoreactive naive B cell activation and the process of somatic hypermutation in germinal center. Due to defective selection process by follicular dendritic cells (FDCs), memory B cells might contribute to plasmacytosis which involved in the development of pathogenic IgG autoantibodies [34, 35]. Altogether, it is possible that the simultaneous expansion of both aNAV and DN2 B cells or T cell-dependent activation of aNAV B cell could contribute to the production of pathogenic autoantibodies that play such a crucial role in SLE pathogenesis. Further investigations are required to clarify their contributions to SLE pathogenesis and the exact mechanisms of activation and differentiation that produce auto-ASCs and whether localized in germinal centers or extra-follicular reactions.

Our study demonstrated that circulating aNAV B cells correlated with increased modified SLEDAI-2 K score, suggesting that these cells’ expansion plays a crucial role in SLE pathogenesis. The aNAV DNA autoreactive B cells, using a tetramer tool, were strongly related to disease activity and anti-dsDNA antibody levels compared with the aNAV B cells within the total B cell population. Using this methodology will be informative in tracking the frequency of aNAV DNA autoreactive B cells in individual patients who develop clinical disease flares.

Reports that heterogeneous autoreactive B cells produce anti-dsDNA antibodies and contribute to SLE pathogenesis have long been a matter of debate. Which specific B cell subset (s) includes DNA autoreactive B cells is still unclear. Using a peptide surrogate (DWEYSVWLSN) for dsDNA and the tetramer tool, we were able to identify and enrich DNA autoreactive B cells that produced anti-dsDNA antibodies. They were mostly accounted for by aNAV B cells, which expressed high surface densities of CD69 and CD86. Interestingly, the expansions of aNAV DNA autoreactive B cells were strongly and positively correlated with modified SLEDAI-2 K scores and anti-dsDNA antibody levels and found in active SLE patients. The explanation is likely that more significant impairment of central tolerance eliminating self-reactive B cells led to the survival of aNAV DNA autoreactive B cells, which then expanded under autoimmune conditions.

Consequently, these expanded aNAV B cells were likely precursors of auto-ASCs and produced the anti-dsDNA autoantibodies central to SLE pathogenesis. The aNAV B cells may be the key players associated with SLE development. However, the study of pathogenic functions of aNAV B cells, such as autoantibody production, pro-inflammatory cytokine secretion, and T cell induction, in the pathogenesis of SLE is required in future studies.

## Conclusion

Our data demonstrated that aNAV B cells could engage with self-antigens expanded under SLE’s autoimmune conditions, and the number of these aNAV B cells was strongly correlated with disease activity. More knowledge in pathogenic function of aNAV B cells is required for development of candidate biomarker for monitoring SLE patients.

## Supplementary Information

**Additional file 1.** Table S1. Demographic characteristics in study subjects for the phenotyping of total B cell subsets.

**Additional file 2.** Table S2. Demographic characteristics in study subjects for the phenotyping of DNA tetramer-binding B cells

**Additional file 3.** Table S3. Correlation analysis of aNAV in total and DNA tetramer-binding B cells with clinical laboratory parameter.

**Additional file 4.** Figure S1. Relative expression of activating B cell surface markers on activated naïve DNA tetramer-binding B cells.

## Data Availability

The datasets used and/or analyzed during the current study are available from the corresponding author upon reasonable request.

## Abbreviations

aNAV: Ativated naïve
Anti-dsDNA: Anti-double stranded DNA
ASCs: Antibody-secreting cells
AtMs: Atypical memory B cells
ABCs: Age-associated B cells
APC: Allophycocyanin
C3: Complement 3
C4: Complement 4
DN1: Double negative 1
DN2: Double negative 2
ESR: Erythrocyte sedimentation rate
FITC: Fluorescein isothiocyanate
FBS: Fetal bovine serum
HBS: HEPES buffer saline
HCs: Healthy controls
Hb: Hemoglobin
IFN-γ: Interferon-γ
IL: Interleukin
IgG: Immunoglobulin G
MS: Master of science
PBMCs: Peripheral blood mononuclear cells
PBS: Phosphate buffer saline
PerCP/Cy5.5: Peridin-chlorophyll protein/Cyanine 5.5
PE: Phycoerythrin
R: Correlation coefficient
R848: Resiquimod
SLE: Systemic lupus erythematosus
modified SLEDAI-2 K: Modified SLE disease activity index-2 K
SLICC: Systemic lupus erythematosus international collaborating clinics
SWM: Switched memory B cells
TLR-7: Toll-like receptor 7
UPCR: Urine Protein to Creatinine
USW: Unswitched memory B cells
WBC: White blood cell count

## References

[1] Raz E, Brezis M, Rosenmann E, Eilat D (1989). Anti-DNA antibodies bind directly to renal antigens and induce kidney dysfunction in the isolated perfused rat kidney. J Immunol (Baltimore, Md: 1950).

[2] Choi J, Kim ST, Craft J (2012). The pathogenesis of systemic lupus erythematosus-an update. Curr Opin Immunol.

[3] Ruchakorn N, Ngamjanyaporn P, Suangtamai T, Kafaksom T, Polpanumas C, Petpisit V, Pisitkun T, Pisitkun P (2019). Performance of cytokine models in predicting SLE activity. Arthritis Res Ther.

[4] Thanadetsuntorn C, Ngamjanyaporn P, Setthaudom C, Hodge K, Saengpiya N, Pisitkun P (2018). The model of circulating immune complexes and interleukin-6 improves the prediction of disease activity in systemic lupus erythematosus. Sci Rep.

[5] Mehra S, Fritzler MJ (2014). The spectrum of anti-chromatin/nucleosome autoantibodies: independent and interdependent biomarkers of disease. J Immunol Res.

[6] Conti F, Ceccarelli F, Perricone C, Massaro L, Marocchi E, Miranda F (2015). Systemic lupus erythematosus with and without anti-dsDNA antibodies: analysis from a large monocentric cohort. Mediat Inflamm.

[7] Davis P, Percy JS, Russell AS (1977). Correlation between levels of DNA antibodies and clinical disease activity in SLE. Ann Rheum Dis.

[8] Isenberg DA, Colaco CB, Dudeney C, Todd-Pokropek A, Snaith ML (1986). The relationship of anti-DNA antibody idiotypes and anti-cardiolipin antibodies to disease activity in systemic lupus erythematosus. Medicine (Baltimore).

[9] Mackworth-Young CG, Chan JK, Bunn CC, Hughes GR, Gharavi AE (1986). Complement fixation by anti-dsDNA antibodies in SLE: measurement by radioimmunoassay and relationship with disease activity. Ann Rheum Dis.

[10] Kavanaugh AF, Solomon DH (2002). Guidelines for immunologic laboratory testing in the rheumatic diseases: anti-DNA antibody tests. Arthritis Rheum.

[11] Adler MK, Baumgarten A, Hecht B, Siegel NJ (1975). Prognostic significance of DNA-binding capacity patterns in patients with lupus nephritis. Ann Rheum Dis.

[12] Swaak AJ, Aarden LA (1979). Statius van Eps LW, Feltkamp TE. Anti-dsDNA and complement profiles as prognostic guides in systemic lupus erythematosus. Arthritis Rheum.

[13] Swaak AJ, Groenwold J, Bronsveld W (1986). Predictive value of complement profiles and anti-dsDNA in systemic lupus erythematosus. Ann Rheum Dis.

[14] Epstein WV (1973). Immunologic events preceding clinical exacerbation of systemic lupus erythematosus. Am J Med.

[15] Mankarious S, Lee M, Fischer S, Pyun KH, Ochs HD, Oxelius VA, Wedgwood RJ (1988). The half-lives of IgG subclasses and specific antibodies in patients with primary immunodeficiency who are receiving intravenously administered immunoglobulin. J Lab Clin Med.

[16] Grimaldi CM, Michael DJ, Diamond B (2001). Cutting edge: expansion and activation of a population of autoreactive marginal zone B cells in a model of estrogen-induced lupus. J Immunol (Baltimore, Md : 1950).

[17] William J, Euler C, Leadbetter E, Marshak-Rothstein A, Shlomchik MJ (2005). Visualizing the onset and evolution of an autoantibody response in systemic autoimmunity. J Immunol (Baltimore, Md : 1950).

[18] Peeva E, Michael D, Cleary J, Rice J, Chen X, Diamond B (2003). Prolactin modulates the naive B cell repertoire. J Clin Invest.

[19] Jacobi AM, Zhang J, Mackay M, Aranow C, Diamond B (2009). Phenotypic characterization of autoreactive B cells--checkpoints of B cell tolerance in patients with systemic lupus erythematosus. PLoS One.

[20] Jenks SA, Cashman KS, Zumaquero E, Marigorta UM, Patel AV, Wang X (2018). Distinct effector B cells induced by unregulated Toll-like receptor 7 contribute to pathogenic responses in systemic lupus erythematosus. Immunity.

[21] Tipton CM, Fucile CF, Darce J, Chida A, Ichikawa T, Gregoretti I, Schieferl S, Hom J, Jenks S, Feldman RJ, Mehr R, Wei C, Lee FEH, Cheung WC, Rosenberg AF, Sanz I (2015). Diversity, cellular origin and autoreactivity of antibody-secreting cell population expansions in acute systemic lupus erythematosus. Nat Immunol.

[22] Wu C, Fu Q, Guo Q, Chen S, Goswami S, Sun S, Li T, Cao X, Chu F, Chen Z, Liu M, Liu Y, Fu T, Hao P, Hao Y, Shen N, Bao C, Zhang X (2019). Lupus-associated atypical memory B cells are mTORC1-hyperactivated and functionally dysregulated. Ann Rheum Dis.

[23] Wang S, Wang J, Kumar V, Karnell JL, Naiman B, Gross PS (2018). IL-21 drives expansion and plasma cell differentiation of autoreactive CD11c^hi^T-bet^+^ B cells in SLE. Nat Commun.

[24] Rodrigues Fonseca A, Felix Rodrigues MC, Sztajnbok FR, Gerardin Poirot Land M, Knupp Feitosa de Oliveira S (2019). Comparison among ACR1997, SLICC and the new EULAR/ACR classification criteria in childhood-onset systemic lupus erythematosus. Adv Rheumatol (London, England).

[25] Batu ED, Kaya Akca U, Pac Kısaarslan A, Sağ E, Demir F, Demir S, et al. SLICC 2012, and EULAR/ACR 2019 classification criteria in pediatric systemic lupus erythematosus. J Rheumatol. 1997;2020. 10.3899/jrheum.200871 33191281

[26] Gaynor B, Putterman C, Valadon P, Spatz L, Scharff MD, Diamond B (1997). Peptide inhibition of glomerular deposition of an anti-DNA antibody. Proc Natl Acad Sci U S A.

[27] Putterman C, Diamond B (1998). Immunization with a peptide surrogate for double-stranded DNA (dsDNA) induces autoantibody production and renal immunoglobulin deposition. J Exp Med.

[28] Kowal C, Degiorgio LA, Lee JY, Edgar MA, Huerta PT, Volpe BT (2006). Human lupus autoantibodies against NMDA receptors mediate cognitive impairment. Proc Natl Acad Sci U S A.

[29] Huerta PT, Kowal C, DeGiorgio LA, Volpe BT, Diamond B (2006). Immunity and behavior: antibodies alter emotion. Proc Natl Acad Sci U S A.

[30] Xin M, Luo EM, Ryan M, O’Connell PW, Yang L, Baltimore D (2009). Engineering human hematopoietic stem/progenitor cells to produce a broadly neutralizing anti-HIV antibody after in vitro maturation to human B lymphocytes. Blood..

[31] Jacobi AM, Reiter K, Mackay M, Aranow C, Hiepe F, Radbruch A, Hansen A, Burmester GR, Diamond B, Lipsky PE, Dörner T (2008). Activated memory B cell subsets correlate with disease activity in systemic lupus erythematosus: delineation by expression of CD27, IgD, and CD95. Arthritis Rheum.

[32] Wei C, Anolik J, Cappione A, Zheng B, Pugh-Bernard A, Brooks J (2007). A new population of cells lacking expression of CD27 represents a notable component of the B cell memory compartment in systemic lupus erythematosus. J Immunol (Baltimore, Md : 1950).

[33] Zumaquero E, Stone SL, Scharer CD, Jenks SA, Nellore A, Mousseau B, et al. IFNγ induces epigenetic programming of human T-bet^hi^ B cells and promotes TLR7/8 and IL-21 induced differentiation. Elife. 2019;8. 10.7554/eLife.41641. 10.7554/eLife.41641 PMC6544433 31090539

[34] Bernasconi NL, Traggiai E, Lanzavecchia A (2002). Maintenance of serological memory by polyclonal activation of human memory B cells. Science (New York, NY).

[35] Wellmann U, Letz M, Herrmann M, Angermüller S, Kalden JR, Winkler TH (2005). The evolution of human anti-double-stranded DNA autoantibodies. Proc Natl Acad Sci U S A.

