# Supplementary material for: The expansion of activated naive DNA autoreactive B cells and its association with disease activity in systemic lupus erythematosus patients
Source: Arthritis Res Ther. 2021 Jul 6;23:179. doi: 10.1186/s13075-021-02557-0 (PMC8259008; doi:10.1186/s13075-021-02557-0)
Supplement: Supplementary file 1 — Additional file 1. Table S1. Demographic characteristics in study subjects for the phenotyping of total B cell subsets. [file 13075_2021_2557_MOESM1_ESM.docx]

**Table S1. Demographic characteristics in study subjects for the phenotyping of total B cell subsets.**

| **Category** | **Feature** | **SLE patients (n = 20)** |
| --- | --- | --- |
| Demographic | Age, year (mean ± SD) | 34.25 ± 14.69 |
|  | Gender, male, n (%) | 2/20 (10 %) |
|  | Female, n, (%) | 18/20 (90 %) |
|  | Disease duration, month (#) | 68 (1-250) |
| Laboratory parameters | Modified SLEDAI-2K (#) | 6 (0-20) |
|  | Anti-dsDNA positive No (%) | 10/20 (50.00 %) |
|  | WBC, cells/mm^3^ (#) | 5550 (2200-12600) |
|  | Hb, g/dl (#) | 11.35 (6.80-13.80) |
|  | UPCR (#) | 0.60 (0.08-10.96) |
|  | ESR, mm/h (#) | 31 (3-127) |
|  | Platelet, x 10^3^ cells/mm^3^ (#) | 242 (108-337) |
|  | Serum creatinine (#) | 0.78 (0.38-3.21) |
|  | C3, mg/l (#) | 1015 (370-1300) |
|  | C4, mg/l (#) | 210 (30-530) |
| Clinical manifestations | Vasculitis, n (%) | 1 (5 %) |
|  | Arthritis, n (%) | 0 (0 %) |
|  | Malar rash, n (%) | 1 (5 %) |
|  | Discoid rash, n (%) | 1 (5 %) |
|  | Purtcher retinopathy, n (%) | 1 (5 %) |
|  | Low complement level n, (%) | 4 (20 %) |
|  | Increase DNA binding n, (%) | 5 (25 %) |
| Treatment | Prednisolone, n (%) | 19 (95 %) |
|  | Antimalarial, n (%) | 18 (90 %) |
|  | Cyclophosphamide, n (%) | 10 (50 %) |
|  | Azathioprine, n (%) | 1 (5 %) |
|  | Mycophenolate, n (%) | 9 (45 %) |
|  | Triamcinolone, n (%) | 2 (10 %) |
|  | Methotrexate, n (%) | 1 (5 %) |

Modified SLEDAI-2K: Modified Systemic Lupus Erythematosus Disease Activity Index 2000; Anti-dsDNA: Anti-double stranded DNA antibody; WBC: White Blood Cell counts; Hb: Hemoglobin; UPCR: Urine Protein to Creatinine; ESR: Erythrocyte Sedimentation Rate; C3: Complement 3; C4: Complement 4. # = [median (range)]
