# Supplementary material for: The expansion of activated naive DNA autoreactive B cells and its association with disease activity in systemic lupus erythematosus patients
Source: Arthritis Res Ther. 2021 Jul 6;23:179. doi: 10.1186/s13075-021-02557-0 (PMC8259008; doi:10.1186/s13075-021-02557-0)
Supplement: Supplementary file 2 — Additional file 2. Table S2. Demographic characteristics in study subjects for the phenotyping of DNA tetramer-binding B cells [file 13075_2021_2557_MOESM2_ESM.docx]

**Table S2. Demographic characteristics in study subjects for the phenotyping of DNA tetramer-binding B cells**

| **Category** | **Feature** | **SLE patients (n = 15)** |
| --- | --- | --- |
| Demographic | Age, year (mean ± SD) | 32.07 ± 12.27 |
|  | Gender, male, n (%) | 0/15 (0 %) |
|  | Female, n, (%) | 15/15 (100 %) |
|  | Disease duration, month (#) | 36 (1-247) |
| Laboratory parameters | Modified SLEDAI-2K (#) | 8 (0-14) |
|  | Anti-dsDNA positive No (%) | 10/15 (66.67 %) |
|  | WBC, cells/mm^3^ (#) | 5400 (2500-9500) |
|  | Hb, g/dl (#) | 12.60 (6.80-14.00) |
|  | UPCR (#) | 0.52 (0.08-4.22) |
|  | ESR, mm/h (#) | 33 (2-127) |
|  | Platelet, x 10^3^ cells/mm^3^ (#) | 256 (91-397) |
|  | Serum creatinine (#) | 0.63 (0.51-2.17) |
|  | C3, mg/l (#) | 1030 (510-1270) |
|  | C4, mg/l (#) | 210 (50-530) |
| Clinical manifestations | Vasculitis, n (%) | 1 (6.67 %) |
|  | Arthritis, n (%) | 1 (6.67 %) |
|  | Malar rash, n (%) | 1 (6.67 %) |
|  | Discoid rash, n (%) | 1 (6.67 %) |
|  | Purtcher retinopathy, n (%) | 1 (6.67 %) |
|  | Low complement level n, (%) | 6 (40 %) |
|  | Increase DNA binding n, (%) | 3 (20 %) |
| Treatment | Prednisolone, n (%) | 15 (100 %) |
|  | Antimalarial, n (%) | 14 (93.33 %) |
|  | Cyclophosphamide, n (%) | 4 (26.67 %) |
|  | Azathioprine, n (%) | 3 (20 %) |
|  | Mycophenolate, n (%) | 7 (46.67 %) |
|  | Triamcinolone, n (%) | 0 (0 %) |
|  | Methotrexate, n (%) | 0 (0 %) |

Modified SLEDAI-2K: Modified Systemic Lupus Erythematosus Disease Activity Index 2000; Anti-dsDNA: Anti-double stranded DNA antibody; WBC: White Blood Cell counts; Hb: Hemoglobin; UPCR: Urine Protein to Creatinine; ESR: Erythrocyte Sedimentation Rate; C3: Complement 3; C4: Complement 4. # = [median (range)].
