# Supplementary material for: The expansion of activated naive DNA autoreactive B cells and its association with disease activity in systemic lupus erythematosus patients
Source: Arthritis Res Ther. 2021 Jul 6;23:179. doi: 10.1186/s13075-021-02557-0 (PMC8259008; doi:10.1186/s13075-021-02557-0)
Supplement: Supplementary file 3 — Additional file 3. Table S3. Correlation analysis of aNAV in total and DNA tetramer-binding B cells with clinical laboratory parameter. [file 13075_2021_2557_MOESM3_ESM.docx]

**Table S3. Correlation analysis of aNAV in total and DNA tetramer-binding B cells with clinical laboratory parameter.**

| **Laboratory parameter** | **Normal ranges** | **aNAV B cells** | | | **aNAV DNA tetramer-binding B cells** | | |
| --- | --- | --- | --- | --- | --- | --- | --- |
|  |  | Median (range) n = 20 | *r* | *p-value* | Median (range) n = 15 | *r* | *p-value* |
| Modified SLEDAI-2K | - | 6 (0– 20) | 0.4991 | 0.0251 | 8 (0 – 14) | 0.7548 | 0.0017 |
| ESR levels, mm/h | 0-20 mm/h | 31 (3 – 127) | 0.4063 | 0.0358 | 33 (2 – 127) | 0.8185 | 0.0006 |
| Anti-ds DNA IgG, IU/ml | < 100 IU/ml | 81.50 (10.00 – 800.00) | 0.4384 | 0.0532 | 85.00 (10.00 – 800.00) | 0.6302 | 0.0138 |
| C3 mean, mg/l | 83-177 mg/l | 1015 (370 – 1300) | -0.4844 | 0.0304 | 1030 (510 – 1270) | -0.3136 | 0.2942 |
| C4 mean, mg/l | 15-45 mg/l | 210 (30 – 530) | -0.5753 | 0.008 | 210 (50 – 530) | -0.3333 | 0.3853 |

Modified SLEDAI-2K: Modified Systemic Lupus Erythematosus Disease Activity Index 2000; ESR: Erythrocyte Sedimentation Rate; Anti-dsDNA: Anti-double stranded DNA antibody; C3: Complement 3; C4: Complement 4; r = Correlation coefficient
