# Supplementary material for: The expansion of activated naive DNA autoreactive B cells and its association with disease activity in systemic lupus erythematosus patients
Source: Arthritis Res Ther. 2021 Jul 6;23:179. doi: 10.1186/s13075-021-02557-0 (PMC8259008; doi:10.1186/s13075-021-02557-0)
Supplement: Supplementary file 4 — Additional file 4. Figure S1. Relative expression of activating B cell surface markers on activated naïve DNA tetramer-binding B cells. [file 13075_2021_2557_MOESM4_ESM.docx]

**Figure S1. Relative expression of activating B cell surface markers on activated naïve DNA tetramer-binding B cells.**


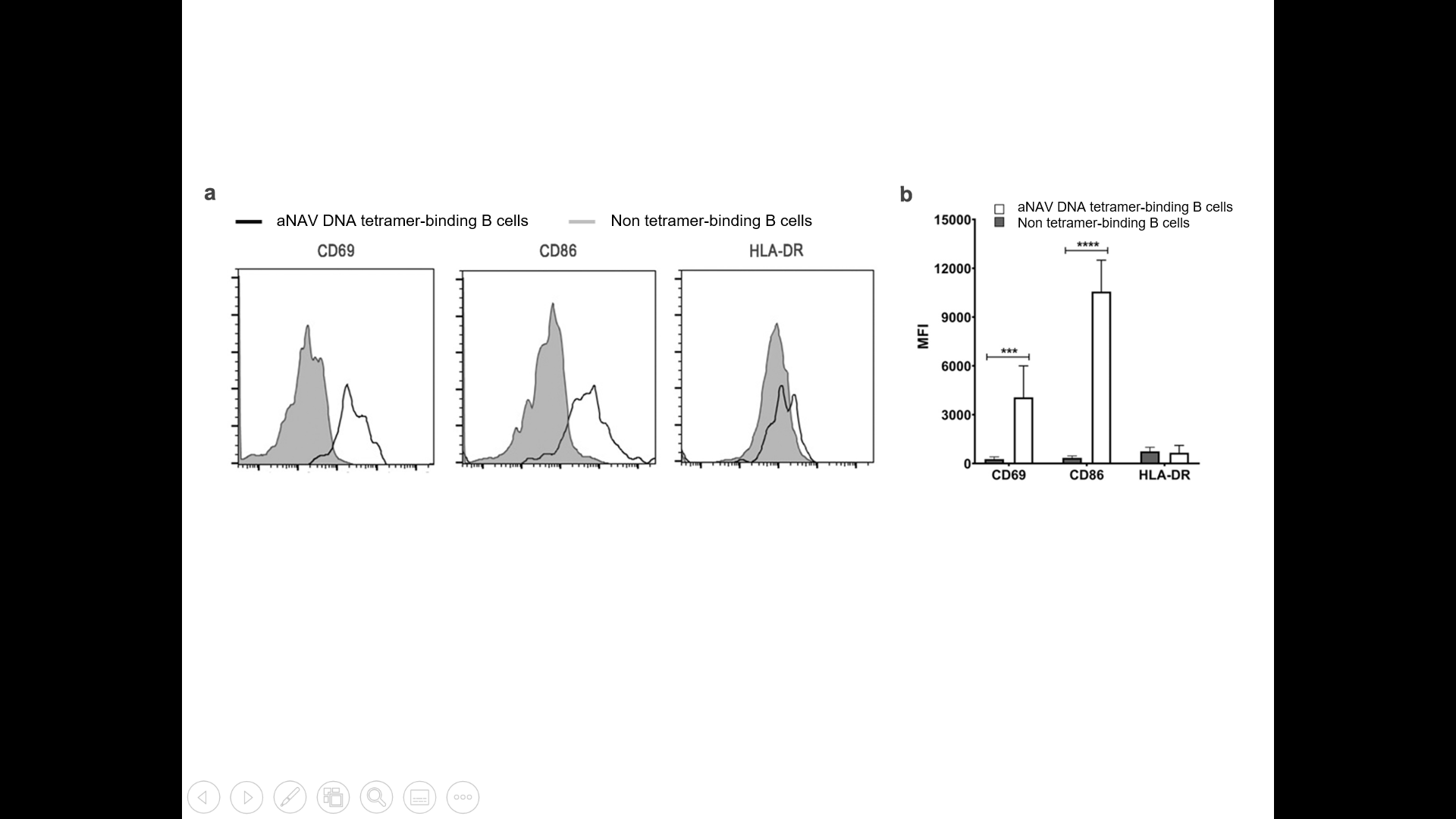


(**a**) aNAV DNA tetramer-binding B cells overexpressed CD69 and CD86 compared to non-tetramer-binding B cells. (**b**) Mean fluorescent intensity (MFI) of CD69, CD86 and HLA-DR on B cells of SLE patients (n = 3). Bars represent median with interquartile range. *p* values were determined by the Mann-Whitney *U*-test: **p* < 0.05; ***p* < 0.01; ****p* < 0.001; *****p* < 0.0001.
